# Clinical and economic impact of coronary artery bypass graft and percutaneous coronary intervention in young individuals with acute coronary syndromes and multivessel disease: A real-world comparison in a middle-income country

**DOI:** 10.3389/fcvm.2022.1000260

**Published:** 2022-11-10

**Authors:** Gustavo de Almeida Alexim, Luiza Ferreira Rocha, Giovani Prediger Dobri, Adair da Silva Rosa Júnior, Ricardo Torres Bispo Reis, Ana Claudia Cavalcante Nogueira, Alexandre Anderson de Souza Munhoz Soares, Andrei Carvalho Sposito, Ana Patricia de Paula, Luiz Sérgio Fernandes de Carvalho

**Affiliations:** ^1^Medical Sciences Post-Graduation Program, Escola Superior de Ciências da Saúde, Brasília, DF, Brazil; ^2^Medical Sciences Post-Graduation Program, University of Brasília, Brasília, DF, Brazil; ^3^Secretaria de Estado de Saúde do Distrito Federal (SES-DF), Brasília, DF, Brazil; ^4^Instituto de Cardiologia e Transplantes do Distrito Federal, Brasília, DF, Brazil; ^5^Department of Mathematics and Statistics, University of Brasília, Brasília, DF, Brazil; ^6^Aramari Apo Institute, Brasília, DF, Brazil; ^7^Department of Cardiology, State University of Campinas (UNICAMP), Campinas, SP, Brazil; ^8^Laboratory of Data for Quality of Care and Outcomes Research, Clarity Healthcare Intelligence, Jundiaí, SP, Brazil; ^9^Gerontology Post-Graduation Program, Universidade Católica de Brasília, Brasília, DF, Brazil

**Keywords:** acute coronary syndromes (ACS), coronary artery bypass graft (CABG), percutaneous coronary intervention (PCI), costs, indirect cost estimation, outcomes assessment

## Abstract

**Background:**

In recent decades, the world watched a dramatic increase in the incidence of acute coronary syndromes (ACS) among young individuals (≤55 years-old) and a relative decrease in the elderly. The management of ACS in young patients with multivessel disease still needs to be elucidated, as these individuals maintain a long life expectancy.

**Research Question:**

To compare clinical outcomes and care costs in individuals with premature ACS and multivessel disease undergoing coronary artery bypass graft surgery (CABG) or percutaneous coronary intervention (PCI).

**Methods and Results:**

Participants included all individuals ≤55 years-old admitted with ACS to public hospitals in Brasília (Brazil) between 2013 and 2015 and who underwent cardiac catheterization with SYNTAX score ≥23 or Duke category 6. Outcomes were adjudicated with death certificates and data from medical records. The primary outcome was the occurrence of major adverse cardiovascular events (MACE), defined as death due to cardiovascular causes, recurrent hospitalizations due to cardiovascular ischemic events, and incident heart failure New York Heart Association III-IV. As secondary outcome we assessed indirect and direct costs by evaluating the cost of lost productivity (in international dollars (Int$) per year) due to illness and death, outpatient costs and costs with new hospitalizations. Multivariate and principal components (PC) adjusted analyzes were performed.

**Results:**

Among 1,088 subjects (111 CABG and 977 PCI) followed for 6.2 years (IQR: 1.1), 304 primary events were observed. MACE was observed in 20.7% of the CABG group and 28.8% of the PCI group (*p* = 0.037). In multivariate analyses, PCI was associated with a hazard ratio (HR) = 1.227 (95% CI: 1.004–1.499; *p* = 0.0457) for MACE, and in PC-adjusted HR = 1.268 (95% CI: 1.048–1.548; *p* = 0.0271) compared with CABG. Despite direct costs were equivalent, the cost due to the loss of labor productivity was higher in the PCI group (Int$ 4,511 (IQR: 18,062)/year vs Int$ 3,578 (IQR: 13,198)/year; *p* = 0.049], compared with CABG.

**Conclusions:**

Among young individuals with ACS and multivessel disease, surgical strategy was associated with a lower occurrence of MACE and lower indirect costs in the long-term.

## Graphical Abstract

In a large cohort of ACS individuals with multi-vessel disease, CABG was related to a lower occurrence of CV deaths or Recurrent ACS as compared to PCI. In addition, comparing the impact of the two strategies, CABG was associated with substantive lower indirect costs. 
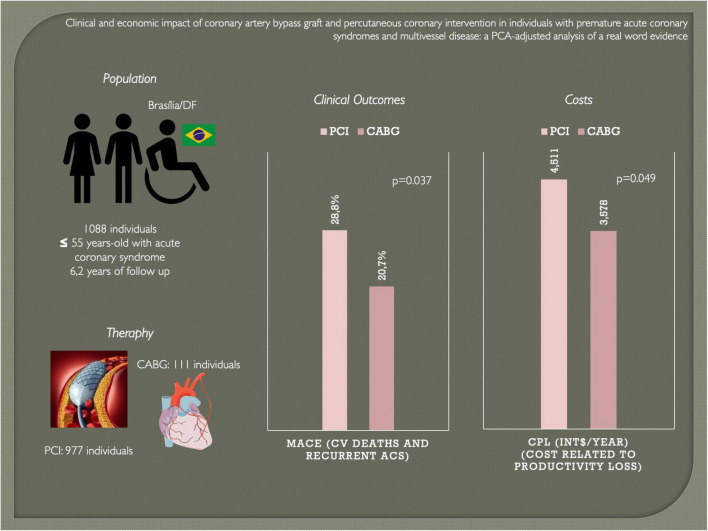

## Highlights

– **Study question:** Over the last decades, young individuals with ACS are more frequently seen with severe multivessel disease. This scenario has dramatic economic impact to society and may result from changes in the epidemiology of chronic disease among young adults. The direct implication of this new scenario is the frequent need to choose between coronary artery bypass graft (CABG) or percutaneous coronary intervention (PCI). In this setting, would CABG or PCI be clinically or economically preferred in young individuals with ACS and multivessel disease?
– **Results:** In a large cohort of ACS individuals, surgical strategy was related to a lower occurrence of major adverse cardiovascular events as compared to PCI, particularly individuals treated with multi-arterial vascular grafts. Indirect costs represent 57% of the economic burden of young individuals with ACS. In addition, comparing the impact of the two strategies, CABG was associated with substantive lower indirect costs.
– **Interpretation:** Our findings suggests that CABG can be the most appropriate revascularization strategy for young individuals who manifest an accelerated and unstable form of coronary artery disease.

## Introduction

Coronary artery disease (CAD) and acute coronary syndromes (ACS) account for the majority of premature deaths in the world (1, 2). Although it occurs predominantly in older individuals, an upward trend in its incidence has been observed in individuals less than 55 years old over last two decades (3, 4).

Although younger individuals with ACS have lower rates of recurrent cardiovascular events when compared to older individuals, the negative impact on the length of life with quality and productivity at work may be greater for younger individuals (5). Symptoms such as angina, dyspnea and depression, commonly found after myocardial infarction (MI), contribute to the decline in quality of life and loss of work capacity at the beginning of professional life (3, 6, 7).

Traditionally, young individuals who manifest MI had a low atherosclerotic burden, often a single-vessel disease (8). In recent years, however, young patients with ACS have presented severe multivessel disease, which may result from changes in the epidemiological profile of the atherosclerotic disease, such as an increase in the prevalence of obesity, metabolic syndrome, diabetes, and drug addiction (9, 10). The direct implication of this change is the frequent need to choose between coronary artery bypass graft (CABG) or percutaneous coronary intervention (PCI) in young patients.

Although guidelines do not indicate the need of changing the revascularization approach according to the age (5), this recommendation is based on data where young individuals are underrepresented (11–13). Taking into account the disparity between younger and older ACS patients in the prevalence of risk factors and in the accelerated development of atherosclerotic disease, it is plausible that there is heterogeneity between them in the response to CABG and PCI (10). If so, it is possible that the choice of therapy without due scrutiny may attenuate the symptom-free life expectancy and even generate a higher direct or indirect cost.

Thus, the present real-world evidence study sought to verify if there is an indication for superiority between PCI and CABG for both clinical evolution (as primary outcome) and costs (as secondary outcome) in young people with ACS and multivessel atherosclerotic coronary artery disease.

## Study design and methods

### Study population

All individuals with a diagnosis of ACS admitted to the Public Healthcare System of Federal District (Brasília, DF, Brazil) and undergoing cardiac catheterization (cath) from January 2013 to December 2015 were included in this retrospective cohort. From 7,392 entries, we found 2,611 entries related to individuals less than 55 years old, of which 1,088 showed moderate to a high atherosclerotic burden, who are at higher risk for cardiovascular events. These individuals were defined by a score that classifies the complexity of CAD, the SYNTAX score, when was ≥23, or a Duke category 6 group (three-vessel severe stenosis of at least 70% or two-vessel severe stenosis with a proximal left anterior descending lesion). The cut-off at 23 for the SYNTAX score is derived from multiple studies conducted with Brazilian individuals with ACS (14, 15), and was used as entry criteria to select a wide group of individuals that could be treated with CABG, not restricted to those with Duke category 6. We excluded individuals who required rescue PCI when STEMI was the index presentation.

The study proceedings are in accordance with the Helsinki Declaration and the study was approved by the Institutional Ethics Review Board (IRB) from *Instituto de Gestão Estratégica do Distrito Federal* (IGESDF) (study protocol approval number [CAAE] 28530919.0.1001.8153). Multivessel PCI procedures were performed either at ‘*Hospital de Base do Distrito Federal*’ or ‘*Instituto de Cardiologia do Distrito Federal*’, as these represented the only facilities capable of performing PCI in individuals hospitalized due to ACS in public hospitals at the Federal District between 2013 and 2015. All CABG surgeries were performed at ‘*Instituto de Cardiologia do Distrito Federal*’.

### Clinical outcomes (primary and secondary outcomes)

The primary outcome was defined as the occurrence of major adverse cardiovascular events (MACE), defined as death due to cardiovascular causes, recurrent hospitalizations due to cardiovascular ischemic events, and incident heart failure New York Heart Association III-IV. The secondary outcomes included the cost of lost productivity (CPL) and years of disease-induced productivity loss (DIYPL).

### Clinical data

Individuals were selected from the list of subjects who had undergone catheterization during study observation window at ‘*Hospital de Base do Distrito Federal*’ and ‘*Instituto de Cardiologia do Distrito Federal*’ cathlabs, institutions that perform nearly 99,8% of all catheterizations of individuals with ACS from the public healthcare system in the region where the study was done. We assessed electronic health record systems and collected data such as demography, and clinical data including information on ACS presentation, past medical history, drugs in use, in-hospital therapies and discharge medications.

### Angiographic data

The analysis of anatomical severity, the extent of coronary atherosclerotic disease, angiographic treatments and left ventricular function were performed by analysis of written reports. The discretion of severity of stenosis adopted was a reduction of arterial lumen ≥70% for epicardial vessels and ≥50% in left main coronary artery. Multivessel disease was characterized by the 3 or more major epicardial vessels with arterial lumen stenosis ≥70% or with left main involvement with stenosis ≥50% (10). The analysis of left ventricular function was defined as preserved in the presence of normal contractility and with dysfunction in the presence of hypokinesia or akinesia.

### Mortality data

The occurrence of deaths was determined by consulting a specific information system for mortality (SIM/SUS), Brazilian Health Ministry. Coding of death certificates in SIM is undertaken to utilize an automated coding system. All deaths require a declaration of cause (death certificate) issued by a physician.

### Cost assessment

Direct costs were assessed with outpatient costs and costs with new hospitalizations during follow-up. We considered the perspective of the Brazilian Unified Health System (SUS) as the payer. The amounts reimbursed for cost items are standardized across the country based on the SUS price list (described in Supplementary Table 2). For Brazilian costs, the monetary values of the SUS price list were obtained in reais (R$) and later converted to international dollars (Int$) considering the purchasing power parity (PPP) (conversion factor 2.36). This method of extracting data from SUS databases was previously described (16).

To assess indirect costs, i.e., costs associated with lost productivity due to illness and death in this population, two variables were used: cost of lost productivity (CPL), and years of disease-induced productivity loss (DIYPL), which are derived from years of potential life lost (YPLL), years of potential productive life lost (YPPLL).

The YPLL measures the difference in life expectancy between patients with a particular clinical condition and the general population (17). Estimates the time the person should have lived if he had not died prematurely. To calculate the YPLL, we first distribute the deaths by age group. We then calculated the mean age of each group and subtracted it from the life expectancy of 65 years. In addition, the number of deaths in each age group is multiplied by the number of years left to reach the age of life expectancy. The sum of these products provides the total number of potential years of life lost due to premature acute coronary syndromes (18).

YPPLL represents the number of years of lost productivity resulting from individuals not being able to participate in the workforce due to their condition (19). The formula for calculating this variable is similar to that of YPLL, but life expectancy is replaced by the retirement age (20) subtracted from the lowest productive age (15 years). In this case, we also use the retirement age of 65 years.

CPL is calculated by multiplying YPPLL by the sum of total estimated income from the age of early death to the age of retirement in individuals with premature ACS. This income was based on the average Brazilian salary in the period corrected for the unemployment rate. It represents the loss of productivity in economic value (21, 22). The mean monthly salary among women in 2014 was BRL 1.000,00 (Int$ 366.30), the mean monthly salary among men in 2014 was BRL 1.664,00 (Int$ 609.52) and the unemployment rate in 2014 was 5%. Finally, the DIYPL represents the gross number of years an individual has been forced out of the workforce due to their condition.

### Statistical analyses

From 175 numeric and categorical variables, data were filtered to remove repeated patient entries, non-informative variables, and missing values. After this filtering step, the dataset consisted of 154 variables. Through the analysis of the Variance Inflation Factor (VIF) performed with the processed data, multicollinearity was detected between many variables. Those and those with strong positive or strong negative correlations (| r| > 0.7) were excluded from the dataset. Clinical analysis was performed to further identify the variables that could be removed from the final models, reducing the total number of variables to 101. Variables were grouped according to context and clinical importance.

Principal Component (PC)-analysis was performed within each group of variables and sets of weights were produced by scaling the scores of each first principal component, chosen in this way to explain a larger percentage of the variance of the data set. Each new composite variable is a linear function of the variables in each group, with weights given by standardizing the PC scores. A total of 8 components were segregated: “demography,” “index ACS,” “comorbidities,” “discharge medications,” “coronary disease severity,” “acute phase catheterization data,” “catheterization procedures/interventions,” “left ventricular (LV) function.” In demography PC, we included gender, age, ethnicity and family income. Index ACS PC, we included the cause of hospitalization in the index event, followed by acute phase treatment variables such as frequency of thrombolysis, primary PCI, pharmacoinvasive strategy and late admission (>12 h). Comorbidities PC included the frequencies of T2DM, T2DM on insulin, smokers, dyslipidemia, hypertension, family history of CAD, obesity, atrial fibrillation and prior CAD. Discharge medications PC included the frequency of nitrate, statin, betablockers, ARB or ACEi, CCB, aspirin (ASA), clopidogrel, prasugrel, ticagrelor, oral anticoagulants (OAC), spironolactone and furosemide. Coronary disease severity PC included Syntax score and the presence of specific patterns of CAD: 1-vessel, 2-vessel, 3-vessel disease and/or the presence of Left main disease. Acute phase catheterization PC included detailed information regarding coronary stenosis in each segment and each artery, including the presence of prior stents, focal stenoses, calcific lesions and thrombus. Catheterization procedures/interventions included detailed information regarding the stent’s length, diameter, whether predilatation and/or postdilatation were used, as well as the type of stents (BMS or DES). LV function PC included LV ejection fraction, LV diameter and the presence of aortic stenosis. PC-adjusted multivariate analyses were used as a resource to reduce the risk of Cox regression overfitting, a problem that arises when too many covariates are included in models. As observed by other authors (23), PC-adjustment was consistently superior to propensity scores in our dataset since it showed improved balance and explained a larger percentage of the variance of the dataset compared to propensity scores.

In this study, the primary objective is to compare the incidence of MACE related to revascularization treatments (CABG vs PCI) in patients with acute coronary syndromes at an early age (≤55 years). The sample size was estimated based on data found in a large study (24) that observed a 6-year incidence of mortality in patients with the multivessel disease treated with CABG of 10.2 vs 17.2% in patients with the multivessel disease treated with PCI. Considering, therefore, that the absolute difference between the groups was 7%, a power (1 - beta) of 90% for a two-tailed alpha of 5%, a total sample size of 1,010 individuals would be sufficient to determine the superiority of one treatment compared to the second.

The distribution of variables and their normality were checked using histograms, scatter plots, and the Kolmogorov–Smirnoff and Shapiro–Wilk tests. For comparison between the groups, we used chi-square test for categorical variables, t-test for continuous variables with normal distribution, and Mann–Whitney test for continuous variables with the non-parametric distribution. To analyze the incidence of death during clinical follow-up, Kaplan–Meier curves were constructed with log-rank tests to compare groups. Cox regressions were used for time-dependent proportional risk factors being constructed using a stepwise rule. Cox regressions were also adjusted using only components derived from the PCs. Data were presented as mean ± standard deviation for normally distributed data and as median [interquartile range (IQR)] for nonparametric data. The value of *p* ≤ 0.05 was considered statistically significant. Statistical analyzes was performed using R Studio v.1.1.463, R language version 4.0.1 for Mac.

## Results

We identified 1,088 subjects eligible to participate in our study, of which 111 subjects (10.2%) underwent CABG and 977 (89.8%) underwent PCI. In both groups, most subjects were men (CABG 70.3% and PCI 64.3). On admission, the most frequent diagnosis was ST-segment Elevation Myocardial Infarction (STEMI) (CABG 43.2% and PCI 48.9%), followed by non-ST-segment Elevation Myocardial Infarction (NSTEMI) (CABG 28.8% and PCI 29.3) and, finally, Unstable Angina (UA) (CABG 27.9% and PCI 21.8%). As for risk factors, comparing CABG and PCI, those that showed a statistically significant difference were dyslipidemia (24.3 vs 15.3%, respectively) and history of previous CAD (9 vs 4.1%). Furthermore, it was observed that most individuals in both groups had hypertension (CABG 69.4% and PCI 60.8%). As expected, the CABG group showed a higher average Syntax score compared to the PCI group (32 vs 24, *p* < 0.001). Finally, most patients evolved with left ventricular dysfunction in PCI compared to CABG (23.6 vs18%, *p* = 0.028).

Most individuals (75%) admitted due to STEMI were treated by chemical thrombolysis, 15% treated by primary PCI and 10% were admitted outside treatment window (late admission). Sixteen percent of those treated by chemical thrombolysis required rescue PCI. In our setting, only 20% of those treated by chemical thrombolysis had access to pharmacoinvasive strategy. The remaining 64% treated by chemical thrombolysis and late admissions underwent elective coronarography after a median of 5 days since hospital admission due to STEMI. At this point, individuals with 3-vessel disease or LM are elected either for CABG or multivessel PCI.

**FIGURE 1:**
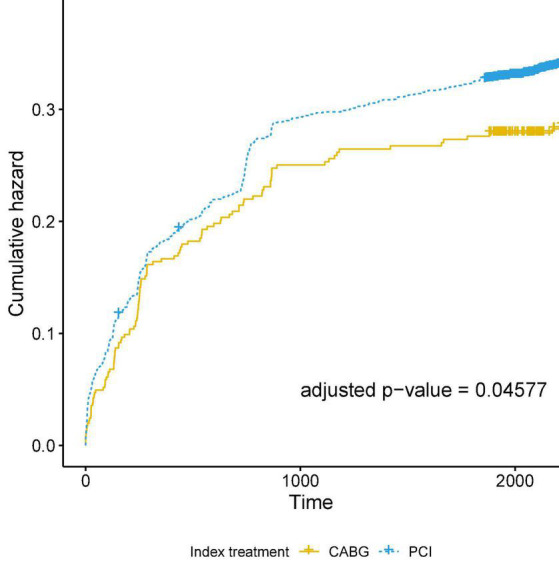
Kaplan–Meier curve for the cumulative hazard for major adverse cardiovascular events (MACE), a compound endpoint of death due to cardiovascular causes, recurrent hospitalizations due to cardiovascular ischemic events and incident heart failure New York Heart Association III-IV.

During a median follow-up of 6.67 years (95% confidence interval [CI] of 5.59–7.24), MACE occurred in 304 individuals. The risk of MACE was significantly higher in the PCI group (28.8 vs 20.7%; *p* = 0.037), with the risk of re-infarction being the event with a statistically significant difference between the PCI and CABG groups, respectively (15 vs 6.3%; *p* = 0.014) (Table 1 and Figure 1). In an adjusted Cox Regression model (Table 2) to assess the occurrence of MACE, we found that the PCI group had a 22.7% higher risk of MACE when compared to the CABG group (95% CI: 1.004 to 1.499; *p* = 0.045).

**TABLE 1:** Clinical and cost outcomes.

	CABG	PCI	*p*
*N*	111	977	
**Clinical outcomes (considering competing risks)**			
Follow-up time, days [median (IQR)]	2,311 (633)	2,348 (524)	0.487
MACE (%)	23 (20.7)	276 (28.2)	0.037
Death	9 (8.1)	64 (6.6)	0.450
MI	7 (6.3)	147 (15.0)	0.014
UA with hospitalization	6 (5.4)	65 (6.7)	0.812
HF NYHA III-IV	1 (0.9)	10 (1.0)	1.000
Readmissions due to stable angina	1 (0.9)	8 (0.8)	1.000
**Cost outcomes**			
Direct costs* [Int$/year, median (IQR)]	3,141 (15,392)	3,348 (17,884)	0.802
Cost of productivity lost [Int$/year, median (IQR)]	3,577 (13,198)	4,511 (18,062)	0.049
Disease-induced years of productivity lost [years, mean (SD)]	1,678 (471)	1,884 (649)	0.001

HF, heart failure; MACE, major adverse cardiovascular events; MI, myocardial infarction; NYHA, New York Heart Association; UA, unstable angina. MACE includes cardiovascular death, recurrent hospitalizations due to cardiovascular ischemic events, and incident heart failure New York Heart Association III-IV. *Direct costs were calculated with values described in Supplementary Table 2 using data for Brazil obtained from DATASUS (SIH/SUS and SIGTAP), the data processing system of the Brazilian Health Ministry.

**TABLE 2:** Cox regression for the occurrence of major adverse cardiovascular events (MACE, cardiovascular death, recurrent hospitalizations due to cardiovascular ischemic events, and incident heart failure New York Heart Association III-IV) as dependent variable.

	HR	95% CI		*p*
		Lower	Upper	
PCI vs CABG	1,227	1,004	1,499	0.04577
Age (each 1 year)	1,013	1,007	1,018	<0.0001
Male gender vs Female	1,053	0.930	1,192	0.41261
STEMI vs NSTEMI	1,011	0.872	1,172	0.88343
STEMI vs UA	1,036	0.887	1,210	0.65638
T2DMvs non-T2DM	1,098	0.964	1,250	0.15805
Prior CAD vs absent CAD	1,261	0.975	1,631	0.07716
Severe CAD (Synthax score > 32)	1,054	0.921	1,206	0.44689
Statin at discharge	1,049	0.889	1,236	0.57007
OAC at discharge	2,157	1,672	2,781	<0.0001
Beta-blocker at discharge	0.765	0.673	0.872	<0.0001
Furosemide at discharge	1,233	1,042	1,458	0.01451
LVEF < 45%	1,266	1,080	1,484	0.00367

CAD, coronary artery disease; CABG, coronary artery bypass graft; LVEF, left ventricular ejection fraction; NSTEMI, non-ST-segment elevation myocardial infarction; OAC, oral anticoagulants; PCI, percutaneous coronary intervention; STEMI, ST-segment elevation myocardial infarction; T2DM, type 2 diabetes mellitus; UA, unstable angina.

The comparison between individuals receiving multi-arterial coronary artery bypass graft (MA-CABG) vs single-arterial CABG suggests MA-CABG confers more robust protection against MACE. MA-CABG showed a 5 vs 24% incidence of MACE (*p* = 0.012).

Regarding the selection of clinical outcomes, one could notice periprocedural MI related to CABG are not included in this analysis. However, it is noteworthy that among 5 individuals in CABG group who had periprocedural MI (4.5%), all died during index hospitalization and these deaths were counted. These events occurred during a generally long hospital stay after ACS (time interval from ACS admission to post-CABG discharge between 22 and 139 days). This long time takes into account an average waiting time for CABG of 55 ± 23 days (range: 17–119 days) and an average hospital stay after CABG of 7 ± 6 days (range: 5–61 days) (Table 3).

**TABLE 3:** Characteristics of enrolled individuals.

	CABG	PCI	*p*
*N*	111	977	
**Demography**			
Age [mean (SD)]	49.50 (4.16)	47.74 (5.83)	0.002
Male gender (%)	78 (70.3)	628 (64.3)	0.251
Acute phase presentation and treatment			
Index diagnosis [*n* (%)]			0.310
STEMI	48 (43.2)	478 (48.9)	
NSTEMI	32 (28.8)	286 (29.3)	
UA	31 (27.9)	213 (21.8)	
**STEMI**			
Primary thrombolysis	41 (85.4)	353 (73.8)	0.012
Primary PCI	0 (0)	72 (15.1)	<0.001
Rescue PCI	0 (0)	0 (0)	1.000
Pharmacoinvasive strategy	0 (0)	95 (19.9)	<0.001
Late admission (>12 h) with exclusive clinical treatment in acute phase	7 (14.6)	53 (11.1)	0.316
**Comorbidities**			
T2DM (%)	28 (25.2)	207 (21.2)	0.391
T2DM on insulin (%)	7 (6.3)	55 (5.6)	0.940
Smokers (%)	46 (41.4)	443 (45.3)	0.495
Dyslipidemia (%)	27 (24.3)	149 (15.3)	0.020
Hypertension (%)	77 (69.4)	594 (60.8)	0.098
Family history of CAD (%)	14 (12.6)	176 (18.0)	0.198
Obesity (%)	5 (4.5)	85 (8.7)	0.181
Atrial fibrillation (%)	0 (0.0)	10 (1.0)	0.585
Prior CAD (%)	10 (9.0)	40 (4.1)	0.035
**Discharge meds**			
Nitrate (%)	46 (41.4)	399 (40.8)	0.984
Statin (%)	99 (89.2)	787 (80.6)	0.037
Betablockers (%)	86 (77.5)	629 (64.4)	0.008
ARB or ACEi (%)	39 (60.3)	598 (61.2)	0.280
CCB (%)	20 (18.0)	184 (18.8)	0.936
ASA (%)	83 (74.8)	665 (68.1)	0.181
Clopidogrel (%)	38 (34.2)	681 (69.7)	<0.001
Prasugrel (%)	5 (4.5)	250 (25.6)	<0.001
Ticagrelor (%)	1 (0.9)	43 (4.4)	0.129
Anticoagulant (%)	3 (2.7)	33 (3.4)	0.923
Spironolactone (%)	12 (10.8)	73 (7.5)	0.291
Furosemide (%)	16 (14.4)	79 (8.1)	0.039
**Severe lesions**			
Syntax score [mean (SD)]	32.3 (2.9)	23.9 (5.1)	<0.001
Duke category 6 group (%)	72 (64.9)	259 (26.5)	<0.001
Duke category 5 group (%)	39 (35.1)	586 (60.0)	<0.001
Duke category 4 group (%)	0 (0.0)	132 (13.5)	<0.001
1- or 2-vessel disease (%)	29 (26.1)	802 (82.1)	<0.001
3-vessel disease (%)	69 (62.2)	173 (17.7)	<0.001
Left main disease (%)	13 (11.7)	2 (0.2)	<0.001
**Cardiovascular interventions indicated in index ACS**			
Number of new stents [median (IQR)]	0.00 [0.00, 0.00]	1.00 [1.00, 2.00]	<0.001
BMS-PCI (%)	–	898 (91.9)	
DES-PCI (%)	–	79 (8.1)	
SA-CABG	91 (82.0)	–	
MA-CABG	20 (18.0)	–	
Waiting time for CABG [days, mean (SD)]	55 (23)	–	
Hospitalization time after CABG [days, mean (SD)]	7 (6)		
**Echocardiography**			
LV function at 5th day (%)			0.028
<45%	20 (18.0)	231 (23.6)	
>45%	91 (82.0)	746 (76.4)	

ACEi, angiotensin converting enzyme inhibitor; ARB, angiotensin receptor blocker; ASA, acetylsalicylic acid; BMS, bare metal stent; CAD, coronary artery disease; CCB, calcium channel blocker; DES, drug eluting stent; LV, left ventricular; MA-CABG, multi-arterial coronary artery bypass graft; NSTEMI, non ST-segment elevation myocardial infarction; PCI, percutaneous coronary intervention; SA-CABG, single-arterial coronary artery bypass graft; STEMI, ST-segment elevation myocardial infarction; T2DM, type 2 diabetes mellitus; UA, unstable angina.

We generated 8 principal components (PC) that captured 92% of variance in the dataset based on variable clusters: Demography, Index ACS, Comorbidities, Discharge meds, Coronarography data, CAD severity, LV dysfunction and coronary procedures (differences in each component across PCI and CABG groups are available in Supplementary Table 1). When using PC-adjusted Cox regression for the occurrence of MACE, PCI was also associated with an increased risk compared to CABG with HR of 1.268 (95% CI: 1.048 to 1.548; *p* = 0.027) (Table 4).

**TABLE 4:** Principal component (PC)-adjusted Cox regression for MACE as dependent variable.

	HR	95% CI		*p*
		Lower	Upper	
PCI vs CABG	1,268	1,048	1,548	0.0271
Demography	1,000	1,000	1,000	0.0625
Index ACS	1,039	0.898	1,202	0.6025
Comorbidities	0,781	0.623	0.977	0.0312
Discharge meds	0,834	0.681	1,022	0.0798
Coronarography procedures	0,998	0.990	1,006	0.6500
CAD severity	1,008	0.965	1,052	0.7314
LV dysfunction	0,905	0.827	0.989	0.0292

ACS, acute coronary syndromes; CAD, coronary artery disease; CABG, coronary artery bypass graft; LV, left ventricular; PCI, percutaneous coronary interventions.

Regarding individual components of MACE, only re-infarction was statistically different across groups, occurring in 7 individuals (6.3%) treated with CABG and 147 (15.0%) in the PCI group (*p* = 0.014). The frequency of death, severely symptomatic heart failure and unstable angina with hospitalization were not different across groups. The risk of MACE among individuals who had undergone single-artery CABG (SA-CABG) was not different from those who received multi-arterial CABG (MA-CABG) (*p* = 0.45).

Among secondary outcomes, the overall cost related to productivity loss (CPL) favors surgery with a median CPL of Int$ 3,577[IQR: 13,198] for CABG, against Int$ 4,511[IQR: 18,061] for PCI (*p* = 0.049), although it did not reach statistical significance. However, disease-induced years of productivity lost (DIYPL) was remarkably lower in individuals treated with CABG (mean of 1,678 [SD: 471] years) compared to PCI (1,884 [SD:648] years; *p* = 0.001). Direct costs were not different between CABG and PCI with median of Int$ 3,141 [IQR: 15,392] and Int$ 3,348 [IQR: 17,884], respectively (*p* for difference = 0.8). We observed that indirect costs represented 57% and direct costs 43% of the overall economic burden of young individuals with ACS and multivessel disease.

## Discussion

In the present study, we observed that CABG was associated with a decrease both in the long-term incidence of MACE and indirect costs related to productivity loss in young individuals with ACS and multivessel disease as compared to individuals treated with PCI. Similar findings were also described in older subjects (24–26), particularly favorable in those receiving multiple arterial bypass grafts (24). The major novelty of our study is to bring a clear perspective in terms of the economic impact of ACS in young adults and to show that widening access to CABG could mitigate indirect costs related to productivity losses.

The availability of CABG worldwide has decreased over the last 15 years with the PCI to CABG ratio increasing (27, 28). This changing landscape is associated with the good short-term results of PCI, as well as advances in PCI techniques and armamentarium with drug-elution stents, intravascular ultrasounds, etc. In parallel, CABG usually demands more medical resources and favors longer periods of hospitalization (27). Consistently, in our study, the vast majority of patients underwent PCI (89.8 vs 10.2% treated with CABG) (9:1 PCI to CABG ratio). These findings are also true for older subjects in our cohort with a 7:1 PCI to CABG ratio (data not shown). This proportion is in agreement with several studies (25–28), whose PCI to CABG ratio ranges from 8:1 to 20:1 among young individuals and 6:1 to 9:1 among older subjects.

The therapeutic decision regarding PCI vs CABG is typically influenced by the patient’s age and the relative availability of both procedures (27). Younger age usually favors the indication of PCI compared to CABG (26). In line with this, 26% of subjects in our cohort who should have been treated by CABG (Duke category 6) were treated with PCI, suggesting a bias toward PCI. In this context, when CABG is indicated in a patient hospitalized with ACS in the public health system in Brazil, the average waiting time for CABG frequently exceeds 60 days in many regions (29). So, there is a natural trend for selecting PCI to reduce the length of hospitalization. This context could also overlay scenarios where an incorrect therapeutic choice would induce worse results with PCI compared to CABG.

Although young individuals with ACS usually present a lower recurrence of MACE and lower direct costs as compared to older subjects (9), our study shows that the indirect economic impact of ACS in young subjects is robust, with disease-induced loss in productivity 12% higher (*p* < 0.001) in the PCI group compared to CABG. It means that addressing young individuals with the best care and optimizing the control of risk factors are potential solutions to reduce the overall socio-economic impact of ACS. In face of our results, this landscape suggests that expanding access to CABG may potentially reduce productivity losses and indirect costs attributed to ACS.

A number of definitions of early-onset CAD have been described in literature. We followed the definition used by Jeemon et al. (30) and Zeitouni et al. (31), which is widely used in clinical trials and supported by a clear break in cardiovascular risk thresholds seen in individuals younger than 55 years old compared to older subjects (32).

Some limitations must be acknowledged. This is a retrospective study and this could influence the associations we found through a potential lack of information biased toward PCI or CABG groups. We mitigated this potential limitation by using only official data records to ascertain deaths or recurrent ischemic events. As we performed the study in all public hospitals that perform PCI or CABG in a whole district and the crossover between public and private sectors is minimal, we also reduced the risk of selection bias. However, as clinical guidelines suggest 3-vessel disease or left main disease should be treated by CABG, an indication bias may not be circumventable. It is noteworthy that the marked class imbalance (90% of individuals were treated with PCI and only 10% with CABG) can raise the risk of Type II error, and suggests that negative findings (such as the absent difference between PCI and CABG groups in terms of direct costs) could be the product of relatively low statistical power. However, it is important to mention that this cohort of young individuals with ACS is among the largest of its kind.

Multivessel PCI procedures used mostly bare-metal stents (BMS), which are associated with higher rates of restenosis, 16% increased risk for myocardial infarction in the first year and no change in the risk of death compared to drug-eluting stents (DES) (33). The decision regarding BMS and DES was based on the availability of each device in Brasília’s Public Health System between 2013 and 2015. If the real-world practice in Brasília included only multivessel PCI with DES rather than BMS, direct and indirect costs in PCI group would be slightly reduced.

It is also important to mention that it is unclear whether these results could be extrapolated to the United States (US) or European countries. There is a wide variation in the cost of CV events within and across countries, which are attributed to differences in study populations, costing methods, and reporting differences. CABG cost in the US averaged $57,577 with a range of cost estimates from $17,731 to $124,221; and in Europe $14,562 with a range of costs from $924 to $27,000. PCI cost in the US is estimated at $20,146, which are nearly one-third of the average CABG costs. Average CABG costs in Europe, however, are only slightly higher than PCI costs (34). The PCI:CABG cost ratio in Brazil is closer to the US than that seen in Europe.

Unfortunately, in this study procedural covariates were not available for CABG, except if multiarterial or single-arterial grafts were used. As such, it is not possible to evaluate the impact of exact types of grafts used, vessels treated, completeness of revascularization or the use of “off-pump” strategy. Although lesion length, diameter and morphology are not available in our dataset, the SYNTAX score was priorly calculated and that takes into account such variables to define the anatomical complexity. Ultimately, unmeasured or unknown factors could play a role in the results as the treatment choices were made by clinical teams at the discretion of study investigators. The confirmation of these results by a randomized controlled trial is required in order to guide clinical practice.

Huge advances have been made with improvements in both PCI and CABG techniques. Particularities even favor the combination of the two approaches in certain clinical settings. Our present findings open the room for debating suggesting that CABG can be the most appropriate revascularization strategy for young individuals who manifest an accelerated and unstable form of coronary artery disease.

## Data availability statement

The data analyzed in this study is subject to the following licenses/restrictions: All requests for raw and analyzed data and related materials, excluding programming codes, will be reviewed by the Clarity Healthcare Intelligence legal department to verify whether the request is subject to any intellectual property or confidentiality obligations. Requests for patient-related data can be considered upon request. Any data and materials that can be shared will be released via a Material Transfer Agreement. Requests to access these datasets should be directed to clarityhealth21@gmail.com.

## Ethics statement

The studies involving human participants were reviewed and approved by Comitê de Ética em Pesquisa do Instituto de Gestão Estratégica em Saúde do Distrito Federal. Written informed consent for participation was not required for this study in accordance with the national legislation and the institutional requirements.

## Author contributions

LC and GA: concept and design and provision of study materials or patients. LC, GA, LR, GD, and AR: acquisition of data and drafting of the manuscript. LC, RR, and GA: analysis and interpretations of data. ACS, AS, LC, GA, AN, and AP: critical revision of the manuscript for important intellectual content. LC and RR: statistical analysis. LC: obtaining funding. AN, GA, and AS: administrative, technical, or logistic support. LC and AP: supervision. All authors contributed to the article and approved the submitted version.

## Abbreviations

ACEi: angiotensin converting enzyme inhibitor
ACS: acute coronary syndromes
ARB: angiotensin receptor blocker
ASA: acetylsalicylic acid
BMS: bare metal stent
CAD: coronary artery disease
CABG: coronary artery bypass graft surgery
CCB: calcium channel blocker
CI: confidence interval
CPL: cost of productivity lost
DIYPL: years of disease-induced productivity loss
DES: drug eluting stent
Int$: international dollars
LV: left ventricular
MA-CABG: multi-arterial coronary artery bypass graft
MACE: major adverse cardiovascular events
NSTEMI: non-ST-segment elevation myocardial infarction
PCI: percutaneous coronary intervention
SA-CABG: single-arterial coronary artery bypass graft
SD: side deviation
STEMI: ST-segment elevation myocardial infarction
T2DM: type 2 diabetes mellitus
UA: unstable angina
YPLL: years of potential life lost
YPPLL: years of potential productive life lost.
