# Supplementary material for: Clinical and economic impact of coronary artery bypass graft and percutaneous coronary intervention in young individuals with acute coronary syndromes and multivessel disease: A real-world comparison in a middle-income country
Source: Front Cardiovasc Med. 2022 Nov 10;9:1000260. doi: 10.3389/fcvm.2022.1000260 (PMC9685999; doi:10.3389/fcvm.2022.1000260)
Supplement: Supplementary file 1 [file Table_1.docx]

**Supplementary Material**

**Clinical and economic impact of coronary artery bypass graft and percutaneous coronary intervention in young individuals with acute coronary syndromes and multivessel disease: a real-world comparison in a middle-income country**

Gustavo de Almeida AleximMD, MSc, PhDc^a,b,d^,Luiza Ferreira RochaMD^e^, Giovani PredigerDobriMD^e^,Adair da Silva Rosa JúniorMD^d^, Ricardo Torres Bispo Reis, *Stat*^f^,

Ana Claudia Cavalcante NogueiraMD, MSc, PhDc^b,d,g^,Alexandre Anderson SoaresMD, PhD^g^,

Andrei Carvalho SpositoMD,PhD^g,h^, Ana Patricia de Paula MD, PhD^a^,

Luiz Sérgio Fernandes de Carvalho MD, MSc, PhD^a,b,g,i,j,*^

a. Medical Sciences Post-GraduationProgram, Escola Superior de Ciências da Saúde, Brasília, DF, Brazil

b. Medical Sciences Post-GraduationProgram, Universityof Brasília, Brasília, DF, Brazil

d. Secretaria de Estado de Saúde do Distrito Federal (SES-DF), Brasília, DF, Brazil

e.Instituto de Cardiologia e Transplantes do Distrito Federal, Brasília, DF, Brazil

f. Department of Mathematics and Statistics, University of Brasília, Brasília, DF, Brazil

g. Aramari Apo Institute, Brasília, DF, Brazil

h. Cardiology Department, State University of Campinas (Unicamp), Campinas, SP, Brazil

i. Laboratory of Data for Quality of Care and Outcomes Research,

Clarity Healthcare Intelligence, Jundiaí, SP, Brazil

j. GerontologyPost-GraduationProgram, Universidade Católica de Brasília, Brasília, DF, Brazil

***Correspondingauthor**

Dr Luiz Sérgio Fernandes de Carvalho, M.D. M.Sc., Ph.D.

Laboratory of Data for Quality of Care and Outcomes Research (LaDa:QCOR)

Clarity Healthcare Intelligence

13084-971, Jundiaí, SP, Brazil

E-mail: [luizsergiofc@gmail.com](mailto:luizsergiofc@gmail.com)

**e-Table 1**. Differences between CABG and PCI groups across the 8 principal components

|  | | CABG | PCI | p | |
| --- | --- | --- | --- | --- | --- |
| N | | 111 | 977 |  | |
|  | |  |  |  | |
| Principal components | |  |  |  | |
| Demography (mean (SD)) | 1.95 (0.37) | 1.89 (0.38) | | 0.125 | |
| Index ACS (mean (SD)) | 1.82 (0.84) | 1.93 (0.83) | | 0.199 | |
| Comorbidities (mean (SD)) | 0.26 (0.27) | 0.27 (0.28) | | 0.840 | |
| Discharge meds (mean (SD)) | 0.57 (0.28) | 0.57 (0.31) | | 0.930 | |
| Coronarography data (mean (SD)) | 11.09 (7.31) | 10.64 (8.10) | | 0.579 | |
| CAD severity (mean (SD)) | -0.19 (1.48) | -0.10 (1.45) | | 0.528 | |
| LV dysfunction (mean (SD)) | 0.97 (0.59) | 0.99 (0.69) | | 0.854 | |
| Catheterization procedures (mean (SD)) | 0.80 (1.78) | 0.89 (1.99) | | 0.618 | |
