# Supplementary material for: Clinical and economic impact of coronary artery bypass graft and percutaneous coronary intervention in young individuals with acute coronary syndromes and multivessel disease: A real-world comparison in a middle-income country
Source: Front Cardiovasc Med. 2022 Nov 10;9:1000260. doi: 10.3389/fcvm.2022.1000260 (PMC9685999; doi:10.3389/fcvm.2022.1000260)
Supplement: Supplementary file 2 [file Table_2.docx]

**Supplementary Material**

**Clinical and economic impact of coronary artery bypass graft and percutaneous coronary intervention in young individuals with acute coronary syndromes and multivessel disease: a real-world comparison in a middle-income country**

Gustavo de Almeida AleximMD, MSc, PhDc^a,b,d^,Luiza Ferreira RochaMD^e^, Giovani PredigerDobriMD^e^,Adair da Silva Rosa JúniorMD^d^, Ricardo Torres Bispo Reis, *Stat*^f^,

Ana Claudia Cavalcante NogueiraMD, MSc, PhDc^b,d,g^,Alexandre Anderson SoaresMD, PhD^g^,

Andrei Carvalho SpositoMD,PhD^g,h^, Ana Patricia de Paula MD, PhD^a^,

Luiz Sérgio Fernandes de Carvalho MD, MSc, PhD^a,b,g,i,j,*^

a. Medical Sciences Post-GraduationProgram, Escola Superior de Ciências da Saúde, Brasília, DF, Brazil

b. Medical Sciences Post-GraduationProgram, Universityof Brasília, Brasília, DF, Brazil

d. Secretaria de Estado de Saúde do Distrito Federal (SES-DF), Brasília, DF, Brazil

e.Instituto de Cardiologia e Transplantes do Distrito Federal, Brasília, DF, Brazil

f. Department of Mathematics and Statistics, University of Brasília, Brasília, DF, Brazil

g. Aramari Apo Institute, Brasília, DF, Brazil

h. Cardiology Department, State University of Campinas (Unicamp), Campinas, SP, Brazil

i. Laboratory of Data for Quality of Care and Outcomes Research,

Clarity Healthcare Intelligence, Jundiaí, SP, Brazil

j. GerontologyPost-GraduationProgram, Universidade Católica de Brasília, Brasília, DF, Brazil

***Correspondingauthor**

Dr Luiz Sérgio Fernandes de Carvalho, M.D. M.Sc., Ph.D.

Laboratory of Data for Quality of Care and Outcomes Research (LaDa:QCOR)

Clarity Healthcare Intelligence

13084-971, Jundiaí, SP, Brazil

E-mail: [luizsergiofc@gmail.com](mailto:luizsergiofc@gmail.com)

**e-Table 2.** Mean cost for cardiovascular procedures and hospitalizations during the period from 2013 to 2015 in Brazilian SUS

* Cost of hospitalization (includes lab and imaging exams and the cost for beds during average length of hospital stay), not including the costs for procedures;

** Includes the costs for procedures and hospitalizations. Data for Brazil obtained from DATASUS (SIH/SUS and SIGTAP), the data processing system of the Brazilian Health Ministry.

We used the reimbursement costs for *procedures*,

*ICU hospitalizations* and *in-patient care* to estimate mean annual cost related to relevant clinical events (RECE) including hemodialysis and their relative incidences in patients-years observed in B-CaRe:QCO
